# Knowledge of HbA1c and LDL‐C treatment goals, subjective level of disease‐related information and information needs in patients with atherosclerotic cardiovascular disease

**DOI:** 10.1002/clc.23948

**Published:** 2022-11-30

**Authors:** Maximilian Brockmeyer, Emilia Wies, Jamuna Joerges, Jana Sommer, Sandra Olivia Borgmann, Nadja Chernyak, Yingfeng Lin, Claudio Parco, Volker Schulze, Yvonne Heinen, Malte Kelm, Andrea Icks, Stefan Perings, Georg Wolff

**Affiliations:** ^1^ Division of Cardiology, Pulmonology and Vascular Medicine, Department of Internal Medicine Medical Faculty and University Hospital Düsseldorf, Heinrich Heine University Düsseldorf Düsseldorf Germany; ^2^ Institute for Health Services Research and Health Economics, German Diabetes Center, Leibniz Center for Diabetes Research at the Heinrich Heine University Düsseldorf Düsseldorf Germany; ^3^ German Center for Diabetes Research, Partner Düsseldorf, München‐Neuherberg Germany; ^4^ Institute for Health Services Research and Health Economics, Centre for Health and Society, Medical Faculty and University Hospital Düsseldorf Heinrich Heine University Düsseldorf Düsseldorf Germany; ^5^ CARID – Cardiovascular Research Institute Düsseldorf Germany

**Keywords:** glycated, hemoglobin (HbA1c), information needs, low‐density lipoprotein cholesterol (LDL cholesterol), patient knowledge, patient information, treatment goals

## Abstract

**Background/Hypothesis:**

Risk factor control of diabetes mellitus (DM) and especially dyslipidemia remains unsatisfactory in patients with atherosclerotic cardiovascular disease (ASCVD). We aimed to analyze the knowledge of low‐density lipoprotein cholesterol (LDL‐C) and glycated hemoglobin (HbA1c) treatment goals, subjective level of information, and information needs in very high‐risk patients with ASCVD.

**Methods:**

ASCVD patients (*n* = 210; 75 ± 9 years; 71.4% male; 89.5% coronary disease) with DM (96.7% type 2) completed a questionnaire assessing knowledge of HbA1c and LDL‐C treatment goals and subjective level of information and information needs on disease‐related topics of DM and ASCVD. Serum LDL‐C and HbA1c were measured.

**Results:**

HbA1c goal (<7.0% in 60.6%) was attained more frequently than LDL‐C goal (<70 mg/dl in 39.9%; *p* < .01). Significantly more participants named the correct goal for HbA1c compared to LDL‐C (52.9% vs. 2.4%; *p* < .01). Subjective levels of information were higher and information needs were lower for DM than for ASCVD (*p* < .01 for all topics). No associations of knowledge of treatment goals and level of information with the attainment of treatment goals for HbA1c and LDL‐C were found. However, in multivariate regression, higher levels of education were associated with knowledge of treatment goals (HbA1c: odds ratio [OR] 1.32, 95% confidence interval [CI] 1.01–1.72, *p* = .04; LDL‐C: OR 2.32, 95% CI 1.07–5.03; *p* = .03).

**Conclusion:**

In very high‐risk patients with ASCVD, a deficit of knowledge of treatment goals to control dyslipidemia exists when compared to DM, patients felt significantly better informed for topics of DM than for ASCVD and display higher information needs for topics of ASCVD.

## 1 INTRODUCTION

Secondary prevention of atherosclerotic cardiovascular disease (ASCVD) by modification of risk factors is well‐established to slow disease progression and improve outcomes.^1^ Recommendations in cardiovascular society guidelines (e.g., of the European Society of Cardiology [ESC]) promote pharmacological control of risk factors of dyslipidemia and diabetes mellitus (DM)—among others—and offer precise treatment goals for low‐density lipoprotein cholesterol (LDL‐C) and glycated hemoglobin (HbA1c),^2^, ^3^ which have proven efficient and cost‐effective.^4^, ^5^ However, treatment goal attainment with lipid‐lowering therapy remains poor in clinical trials^6^ and real‐world settings,^7^, ^8^ considerably worse compared to glycemic control in DM.^6^ Cardiovascular societies thus urge physicians to improve guideline implementation in secondary prevention of ASCVD—particularly in lipid‐lowering therapy.^9^

In patients with DM, some evidence exists that better knowledge of disease management goals has been associated with better outcomes, including glycemic control.^10^, ^11^, ^12^, ^13^ Thus far, little is known about the potential role of patient knowledge of treatment goals of lipid‐lowering therapy in cardiovascular disease, urging a further exploration and a comparison of dyslipidemia to DM.^14^, ^15^, ^16^

In patient‐centered health care, there are several interacting factors that can influence this level of implementation.^17^ Physicians are challenged to indicate, inform about, prescribe, and maintain optimal therapy, while patients need to understand and use the information to manage and adhere to it. Recently, updated guidelines on cardiovascular disease prevention in clinical practice^18^ give the highest class of recommendation to an informed discussion about cardiovascular disease risk and treatment benefits tailored to the needs of patients. An essential condition for this discussion is the assessment of disease‐related patient knowledge and information needs. Generally, information is understood as acquired through external sources, whereas knowledge is processed information that gains meaning in individual thinking.^19^ Within the context of health, patient knowledge can be acquired through information provided by physicians or other health professionals as well as other resources.^19^

A perceived deficiency in disease‐related knowledge is thought to raise information needs which might influence patients' behavior regarding treatment decisions.^19^ For instance, patients with diabetes, especially those with comorbidities, have a high need for information,^20^, ^21^ which may also influence secondary prevention efforts on the patient side of ASCVD.^22^, ^23^ Understanding patient information needs might help to identify potential starting points for strategies to improve patient knowledge (e.g., of treatment goals) and thereby secondary prevention of ASCVD.

We thus aimed to analyze objective patient knowledge of treatment goals of lipid‐lowering and antihyperglycemic therapy as well as the subjective level of disease‐related information and information needs in very high‐risk patients with ASCVD. Furthermore, we intended to explore associations of objective knowledge of treatment goals as well as the subjective level of disease‐related information with the respective attainment of treatment goals. Associated factors of knowledge of treatment goals were intended to be identified by multivariate analysis.

## 2 METHODS

### 2.1 Study design, screening, and patient selection

We conducted a cross‐sectional study (German Clinical Trials Register study‐ID: DRKS00019903) between May 2019 and April 2020 in hospitalized patients with known ASCVD that have previously been diagnosed with DM. It was positively evaluated by the ethics committee of the Medical Faculty of Heinrich‐Heine‐University Düsseldorf (Study‐No. 2019‐401‐KFogU) and conducted in accordance with the ethical standards laid down in the Declaration of Helsinki.

All patients ≥18 years of age, hospitalized at Düsseldorf heart center in Germany for any cardiovascular disorder and classified *very high* cardiovascular risk (ESC)^2^, ^3^ due to chronic condition of ASCVD that have previously been diagnosed with DM were eligible for inclusion after written informed consent. Patients with suspected or diagnosed cognitive impairment as well as patients with a language barrier were excluded.

### 2.2 Data assessment and treatment goal definition

Demographic characteristics, comorbidities, history of cardiovascular events, and prescribed lipid‐lowering and antihyperglycemic therapy were identified in medical records and collected into a dedicated database for further evaluation. Self‐reported participation preferences in medical decision‐making were assessed using the Control Preference Scale and coded by *passive role, collaborative role*, and *active role*.^6^ In addition, the highest educational degree was recorded as reported by patients. Peripheral venous blood was sampled to assess LDL‐C and HbA1c serum levels.

Attainment of treatment goals was defined according to 2016 ESC guidelines for the management of dyslipidemia: LDL‐C < 70 mg/dl and HbA1c < 7.0%.^2^ An LDL‐C treatment goal of <55 mg/dl according to the 2019 ESC guidelines was additionally evaluated.^3^

### 2.3 Questionnaire development and utilization

A questionnaire in the German language was composed of a multidisciplinary team of researchers in cardiology, psychology, health communication, and health services research. It was reviewed by external specialists in lipidology and diabetology. Two preliminary versions of the questionnaire were tested for feasibility in 31 patients, resulting in minor changes in layout and composition of questions for the subjective level of information and information needs. The first 50 participants answering the final questionnaire received additional questions for process evaluation, regarding the comprehensibility of the questionnaire; no changes were necessary thereafter. The questionnaire was handed out to participants during their hospital stay and was collected the same day.

To measure objective knowledge, patients were asked to name their treatment goals for HbA1c and LDL‐C or could answer *I don't know*. Correct treatment goals were defined according to ESC guidelines for the management of dyslipidemia,^2^, ^3^ to account for a change of recommended treatment goals during the ongoing study period,^2^, ^3^ both older and newer LDL‐C goals were defined as correct (2016: LDL‐C < 70 gm/dl and HbA1c < 7.0%; 2019: LDL‐C < 55 mg/dl and HbA1c < 7.0%).^2^, ^3^ Furthermore, to assess objective knowledge of the current serum level of LDL‐C and HbA1c patients were asked to state the result of the last measurement or could answer *I don't know*.

Subjective level of information and information needs were assessed with an adapted version of the Information Needs in Diabetes Questionnaire aiming at DM and ASCVD.^24^ Predefined disease‐related topics were selected: *Cause of the disease, course of the disease, long‐term complications, treatment/therapy, lifestyle adjustment, health promotion and information sources (lifestyle adjustment, etc.)*, and *support, helplines, and information sources*. All topics were asked separately for conditions of DM and ASCVD. The subjective level of information was evaluated on a 4‐point Likert scale (*very well, well, not well*, and *not informed at all*). In addition, patients were asked to state a need for additional information on every topic (*yes* or *no*). The questionnaire translated into the English language is available in Supporting Information Materials.

### 2.4 Statistics

Regarding the lack of evidence of patient knowledge of LDL‐C and HbA1c treatment goals and level of disease‐related information we conducted an explorative, hypothesis‐generating study and assumed an adequate sample size of approximately *n* = 200. Data analysis was performed using SPSS 23.0 (IBM) and GraphPad Prism 7.0. Continuous data are presented as means ± standard deviation, ordinal/categorical data are presented as counts and percentage of the total. Dichotomous outcomes of paired data were compared using McNemar's test. Results of all six individual 4‐point Likert items were summed for each participant to compare the overall subjective level of disease‐related information between DM and ASCVD using two‐sided paired *t*‐tests; overall information needs (*yes* or *no*) were analyzed likewise.

Contingency analysis of dichotomous outcomes of knowledge and respective attainment of treatment goals was performed using Fisher's exact test. Correlation analysis was carried out using Spearman's test. Possibly associated factors of knowledge of HbA1c and LDL‐C treatment goals were analyzed by binary logistic regression. Variable selection of included sociodemographic factors was based on previous studies reporting associations with patient knowledge of treatment goals.^10^, ^14^, ^15^ Participation preference and summed subjective level of information were additionally included due to the assumption that patients preferring an active role in medical decision‐making might have higher awareness of disease‐related information. Accordingly, summed subjective level of information was selected as a variable to explore an association of objective knowledge and subjective level of information.

Statistically significant differences of any test result were assumed at *p* < .05. Due to limited knowledge about.

## 3 RESULTS

### 3.1 Sample characteristics

A total of 210 hospitalized patients (mean age 75 ± 9 years, 71.4% male) with ASCVD that have previously been diagnosed with DM were included, patient characteristics are reported in Table 1: Coronary artery disease was the most common ASCVD (89.5%), DM type 2 was the most common type of DM (96.7%). Comorbidities associated with cardiovascular risk were frequent: 93.3% had arterial hypertension, 55.7% had chronic kidney disease (estimated glomerular filtration rate ≤60 ml/min), 31.0% had a history of myocardial infarction, and 51.0% previously underwent percutaneous coronary intervention. A lower secondary education degree was most common (69.1%; International Standard Classification of Education level 2); 15.2% had a university degree (International Standard Classification of Education level ≥6).^25^ According to the Control Preference Scale^6^ participants most commonly selected the *passive role* (56.2%) as the preferred role in medical decision‐making (Table 1).

**Table 1:** Patient characteristics of all included patients.

Baseline characteristics	Total (*n* = 210)
Age (years)	75 ± 9
BMI (kg/m^2^)	28.5 ± 5
Male	150 (71.4%)
Arterial hypertension	196 (93.3%)
Chronic kidney disease (eGFR ≤ 60 ml/min)	117 (55.7%)
Coronary artery disease	188 (89.5%)
Myocardial infarction	65 (31.0%)
Percutaneous coronary intervention	107 (51.0%)
Cerebral artery disease	30 (14.3%)
Stroke	21 (14.7%)
Peripheral artery disease	83 (39.5%)
DM	
Type 1	4 (1.9%)
Type 2	203 (96.7%)
Other type	3 (1.4%)
Diabetic polyneuropathy	35 (16.7%)
Diabetic retinopathy	5 (2.4%)
Highest level of education	
University degree	32 (15.2%)
Higher secondary degree	30 (14.3%)
Lower secondary degree	184 (69.1%)
No degree	3 (1.4%)
Patient participation preference	
*Active role*	42 (20.0%)
*Collaborative role*	50 (23.8%)
*Passive role*	118 (56.2%)

*Note*: Data presented as *n* (%) or as mean ± standard deviation, no missings.

Abbreviations: BMI, body mass index; DM, diabetes mellitus; eGFR, estimated glomerular filtration rate.

### 3.2 Treatment characteristics and attainment of HbA1c and LDL‐C and treatment goals

Any lipid‐lowering therapy was taken by 84.3% of patients, 76.7% were on statin prescription; 28.6% received high‐intensity statin therapy (atorvastatin ≥40 mg/day or rosuvastatin ≥20 mg/day), and 6.2% were on a combination of any statin and ezetimibe. 3.3% of patients not achieving LDL‐C treatment goals received a combination of high‐intensity statin and ezetimibe. None of the participants received a proprotein convertase subtilisin/kexin type 9 (PCSK9)‐inhibitor (Table 2). Insulin therapy was carried out in 42.4% of patients with or without additional oral antihyperglycemic treatment; the other patients were treated with lifestyle recommendations with or without oral antihyperglycemic therapy (Table 2). Details on concomitant cardiovascular medication are listed in Table 2.

**Table 2:** Treatment characteristics.

Treatment	Total (*n* = 210)
Diabetes mellitus therapy	
Lifestyle recommendations ± oral antihyperglycemic therapy	210 (100%)
Insulin therapy	89 (42.4%)
Lipid‐lowering therapy	
Any	177 (84.3%)
Any statin	161 (76.7%)
High‐intensity statin	60 (28.6%)
Ezetimibe	3 (1.5%)
Any statin + ezetimibe	13 (6.2%)
PCSK9‐inhibitor	0 (0)
Antiplatelet/anticoagulant therapy	
Any	201 (95.7%)
Aspirin	117 (55.7%)
P2Y12 inhibitor	42 (20.0%)
Oral anticoagulant	95 (45.2%)
ACE‐inhibitor/angiotensin‐receptor blocker	163 (77.6%)
Betablocker	157 (74.8%)

*Note*: Data presented as *n* (%), no missings.

Abbreviations: ACE, angiotensin‐converting enzyme; PCSK9, proprotein convertase subtilisin/kexin type 9.

**Figure 1:**
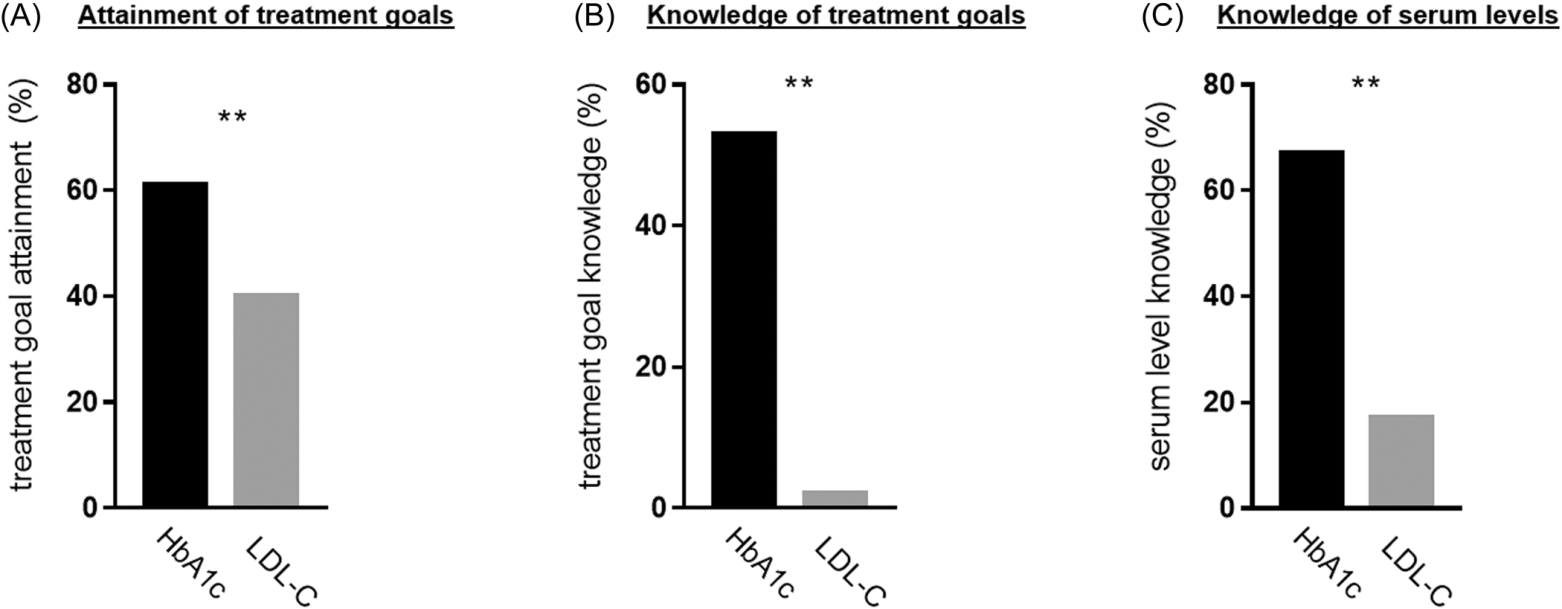
(A) Attainment (*n* = 203) and (B) objective knowledge (*n* = 210) of glycated hemoglobin A1c (HbA1c) and low‐density lipoprotein cholesterol (LDL‐C) treatment goals (in %); (C) knowledge of HbA1c and LDL‐C serum levels, when last measured (in %, *n* = 210); **p < .01.

In 203 of 210 patients, a valid measurement of HbA1c was available; valid LDL‐C values could be retrieved in all patients. All analyses comprising HbA1c and LDL‐C levels including treatment goals were performed in the group of 203 patients. The mean HbA1c was 7.0 ± 1.3% (Figure S1a). Measurement of serum LDL‐C revealed a mean LDL‐C level of 82.9 ± 34.8 mg/dl (Figure S1b). For HbA1c, the treatment goal of <7.0% was attained by 60.6% of patients; the LDL‐C treatment goal of <70 mg/dl was attained by 39.9% of patients (18.7% for LDL‐C goal of <55 mg/dl). The difference in treatment goal attainment was statistically significant (*p* < .01; Figure 1A; Table S1a).

### 3.3 Knowledge of HbA1c and LDL‐C treatment goals and serum levels

All 210 patients answered the complete questionnaire. Analysis of objective knowledge showed 52.9% of patients could name the HbA1c treatment goal correctly, whereas 2.4% could name the correct LDL‐C treatment goal; this difference was statistically significant (*p* < .01; Figure 1B; Table S1b). When asked to name their level of HbA1c and LDL‐C when last measured, significantly more patients reported knowing their last HbA1c: 67.6% stated a value for HbA1c compared to 17.6% for LDL‐C (*p* < .01; Figure 1C; Table S1c).

### 3.4 Subjective level of information and information needs on disease‐related topics

**Figure 2:**
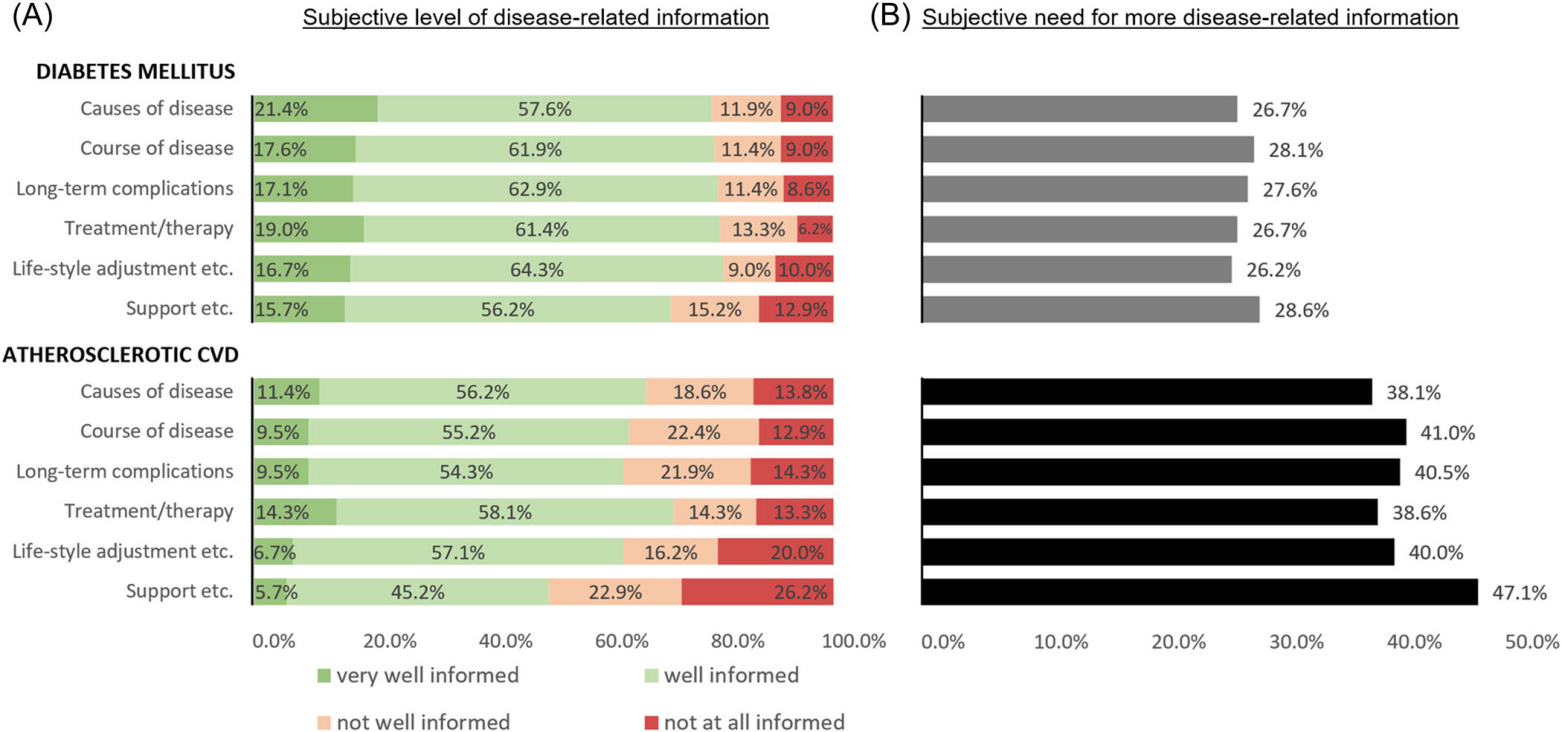
Graphical display of (A) subjective level of disease‐related information on diabetes mellitus (DM) and atherosclerotic cardiovascular disease (ASCVD) and (B) subjective need for more disease‐related information. Summed subjective levels of disease‐related information in DM were significantly higher than in ASCVD (*p* < .01); subjective information needs were lower in DM than in ASCVD (*p* < .01); *n* = 210.

Subjective levels of information and information needs are depicted in Figure 2. For all topics, more than half of the patients felt *very well* or *well informed*. The highest levels of information were found for the topics of *lifestyle adjustment etc*. for DM (81.0% *very well/well informed*) and *treatment/therapy* for ASCVD (72.4% *very well/well informed*). For both DM and ASCVD, the lowest, however, still relatively high levels of information were found for *support, helplines and information sources* (71.9% *very well/well informed* for DM vs. 50.9% for ASCVD). Overall comparison of summed topics showed subjective levels of information were significantly higher for DM than for ASCVD (*p* < .01; Figure 2A).

Information needs were highest for *support, helplines and information sources* for both diseases: 28.6% of patients wished to receive more information for DM and 47.1% for ASCVD. The lowest needs of information were reported for *lifestyle adjustment etc*. for DM (26.2%) and *cause of the disease* for ASCVD (38.1%). Information needs for ASCVD were significantly higher compared to DM in the summed analysis of all disease‐related topics (*p* < .01; Figure 2B).

### 3.5 Associations of knowledge and attainment of treatment goals, HbA1c, and LDL‐C serum levels, and subjective level of information

Further analysis did not identify significant associations of objective knowledge of treatment goal (HbA1c *p* = .09 [two‐sided]; LDL‐C *p* = 1.00 [two‐sided]) and summed subjective level of information (DM ↔ HbA1c *r*_s_ = 0.11; *p* = .13; ASCVD ↔ LDL‐C *r*_s_ = 0.01; *p* = .93) with attainment of treatment goals. No correlation of the summed subjective level of information was found between topics of DM with serum level of HbA1c (*r*_s_ = 0.11; *p* = .14; Figure S2a) and topics of ASCVD with serum level of LDL‐C (*r*_s_ = 0.05; *p* = .47; Figure S2b).

Multivariate logistic regression of possibly associated factors of objective knowledge including age, sex, the highest level of education, participation preference and summed subjective level of information identified the highest level of education as an associated factor of knowledge of HbA1c and LDL‐C treatment goal (HbA1c odds ratio [OR] 1.32, 95% confidence interval [CI] 1.01–1.72; *p* = .04; LDL‐C OR 2.32, CI 1.07–5.03; *p* = .03; Table S2). In addition, higher levels of summed subjective level of information for topics of DM were associated with objective knowledge of HbA1c treatment goal (OR 1.15, CI 1.07–1.24; *p* < .01; Table S2); but no significant association was found of summed subjective level of information for topics of ASCVD with objective knowledge of LDL‐C treatment goal (OR 1.14, CI 0.89–1.47; *p* = .31; Table S2).

## 4 DISCUSSION

We present the results of a cross‐sectional study in 210 patients on knowledge of risk factor control of DM and dyslipidemia in very‐high risk patients with ASCVD. The main results are: (1) knowledge of the HbA1c treatment goal was significantly more frequent compared to knowledge of the LDL‐C goal; (2) subjective level of disease‐related information was significantly higher for DM as compared to ASCVD; (3) patients display higher information needs for topics of ASCVD than for DM; (4) no associations of objective knowledge of treatment goal and subjective level of information with the attainment of treatment goals were found.

### 4.1 Differences in risk factor control of DM and dyslipidemia

Among very‐high cardiovascular risk patients with established ASCVD, risk factor control of DM and dyslipidemia varies: Results of randomized controlled trials in patients with ASCVD and DM, as well as large observational registries show that treatment goal attainment of HbA1c < 7.0% ranges from 45% to 60%.^6^, ^7^, ^26^ In comparison, control of dyslipidemia with precise treatment goals is worse: large European registries revealed that only ∼29%–40% of included patients with ASCVD and DM reached an LDL‐C < 70 mg/dl^7^, ^8^, ^27^; most recent data from EU‐Wide DA VINCI study still showed attainment of LDL‐C goal <70 mg/dl in only 39%, the updated goal of LDL‐C < 55 mg/dl was reached by 18%.^8^ Our findings (40.0% achieving LDL‐C goal of <70 mg/dl and 60.6% achieving HbA1c goal) are thus in line with the current situation in Europe displaying a substantial need for improvement in secondary prevention of ASCVD, especially focusing on lowering of LDL‐C. Regarding differences in risk factor control, the probability of treatment goal attainment with available pharmacotherapy should be considered. Compared to dyslipidemia, in DM type 2 apart from first‐line metformin five other classes of guideline‐recommended oral antihyperglycemic drugs are available to attain an HbA1c goal <7.0% that is above the upper limit of normal.^28^ In contrast, guideline‐recommended oral treatment options to lower LDL‐C < 70 mg/dl, which is by far below the upper limit of normal, are limited to high‐intensity statins and ezetimibe.^3^, ^8^

Complex interactions including patient and physician side contribute to unsatisfactory control of risk factors of dyslipidemia in secondary prevention.^29^ On the physician side, we found a substantial lack of implementation of guideline‐recommended therapy with low rates of LDL‐C treatment attainment in this very high‐risk population: only 35% of patients were on lipid‐lowering therapy with a high‐intensity statin or a combination of statin and ezetimibe. Patients not achieving LDL‐C treatment goals had a combination therapy of high‐intensity statin and ezetimibe in 3.3% only demonstrating the importance of improving guideline adherence among physicians in real‐world settings. Understandably, a lack of optimal therapy as described by the present study as well as the EU‐wide Da Vinci study^8^ is known as an independent risk factor of not achieving treatment goals. Physician characteristics and knowledge is known to be associated with the quality of dyslipidemia management and need to be further investigated and represent possible targets of interventions on the physician side.^30^, ^31^

### 4.2 Patient knowledge and subjective current level of disease‐related information

Comparing risk factors of DM and dyslipidemia, we found substantial differences regarding knowledge of treatment goals, subjective level of information, and information needs, all reflecting patients have higher levels of knowledge and information for DM than for ASCVD and dyslipidemia.

Patient knowledge may be an important modifier of treatment goal attainment: Patients' disease‐related decision‐making and adherence to drug therapy are influenced by adequate patient information.^32^, ^33^ Whereas some evidence exists that patient knowledge of treatment goals is associated with goal attainment of HbA1c in DM,^10^, ^11^, ^12^ the association of LDL‐C treatment goal knowledge and respective goal attainment remains unclear in ASCVD. However, in our study, we could not find associations of objective knowledge or subjective level of information with treatment goal attainment of LDL‐C or HbA1c.

With regard to LDL‐C, only a very small number of patients knew the correct treatment goal, which limited further statistical analyses, and the explorative design of our study does not allow for the identification of causal factors. Recently published results of the GOULD registry on the management of dyslipidemia of ASCVD patients from the United States found ∼10% of patients stated an LDL‐C treatment goal <70 mg/dl, ∼25% in the group treated with a PCSK9‐inhibitor.^15^ Up to this point, GOULD investigators did not publish further analyses of possible associations of knowledge of treatment goals and respective attainment. In our analysis higher levels of education were associated with knowledge of HbA1c and LDL‐C treatment goals; higher levels of education are known to be associated with higher levels of health literacy^34^ and lower rates of cardiovascular events.^35^

Interestingly, we found a discrepancy between objective knowledge of LDL‐C treatment goals and subjective level of information on topics of ASCVD: More than half of the patients felt *very well* or *well informed* for all prespecified disease‐related topics, but only five (2.4%) knew their correct treatment goal for LDL‐C lowering therapy. Patients might have different beliefs about what is important in secondary preventive therapy of ASCVD illustrating the need for improvement in patient‐physician interaction. In addition, disparities in the subjective level of information and objective knowledge between DM and ASCVD in Germany might partially arise from differences in general practitioner‐led disease management programs (DMPs) where the majority of patients with DM as well as patients with coronary artery disease are enrolled: Quality objective defined in DMP of DM includes the attainment of HbA1c goal in more than 60% and a proportion of patients with HbA1c > 8.5% of less than 10%.^36^ No comparable quality objectives could be found in coronary artery DMP until lately the objectives were revised and either a strategy with a high‐intensity statin for all patients or a strategy with the attainment of LDL‐C treatment goal <70 mg/dl has been implemented.^37^, ^38^ Unfortunately, inconsistent LDL‐C treatment goals set up among medical specialties (current ESC goal <55 mg/dl^3^ vs. DMP goal <70 mg/dl^38^) might evoke difficulties in the communication between physician and patients leading to uncertainty or mistrust on the patient side with negative effects, for example, on adherence to lipid‐lowering therapy.

### 4.3 Information needs

In Germany, evidence of information needs in the setting of ASCVD is scarce. The majority of studies were conducted in patients with cancer,^39^ less is known about DM: results from two cross‐sectional studies including approximately 1000 patients^20^, ^21^ show that patients with DM display information needs dependent on, for example, level of information, diabetes therapy, diabetes‐related comorbidity (ASCVD not assessed) and health‐related quality of life. Compared to the study by Borgmann et al., the proportion of people with a need for information on diabetes‐related topics in the present study is similar to or slightly lower.^19^

Our finding of *support, helplines, and information sources* being the topic with the lowest level of information and highest information needs show that especially patients with a need for more support may lack adequate access. Facilitating patients' access to support, helplines, and information sources in the treatment of their ASCVD could be a promising measure to improve patient knowledge and possibly enhance informed discussion about cardiovascular disease risk and treatment benefits in patient–physician interaction. Particular attention should be paid to the availability and access to information based on the criteria of good health information. This will allow patients to receive unbiased, trustworthy, and evidence‐based information to make a decision on their own or together with others.^40^

### 4.4 Limitations

Limitations include the single‐center design and the relatively small sample size, which makes this an explorative study. Furthermore, our study was conducted in a population of old hospitalized patients, which makes results not generally transferable to other populations of ASCVD patients.

We would like to remark that the present study assesses singular factors of a complex construct of interacting factors contributing to the implementation of guideline‐recommended and serves to identify starting points for a better understanding rather than providing a holistic approach.

## 5 CONCLUSION

In very high‐risk patients with ASCVD, a deficit of knowledge of treatment goals to control dyslipidemia exists when compared to DM, patients felt significantly better informed for topics of DM than for ASCVD and patients displayed higher information needs for topics of ASCVD than for DM. Future studies should aim to investigate the influence on patient adherence and identify additional targets to improve the implementation of guideline‐recommended secondary pharmacotherapy, including the healthcare system and physician side.

## AUTHOR CONTRIBUTIONS

Maximilian Brockmeyer, Stefan Perings, Andrea Icks, and Georg Wolff conceived and designed the study. Maximilian Brockmeyer, Emilia Wies, Jana Sommer, Sandra Olivia Borgmann, Andrea Icks, and Georg Wolff primarily designed the questionnaire. Maximilian Brockmeyer, Emilia Wies, and Jamuna Joerges collected data. All authors analyzed and interpreted the data. Maximilian Brockmeyer and Georg Wolff performed the statistical analyses and drafted the manuscript. All authors critically revised the manuscript. All authors read and accepted the submitted version of the manuscript.

## CONFLICT OF INTEREST

The authors declare no conflict of interest.

## Supporting information

Supplementary information.

## Data Availability

All data relevant to this study are included in the article or uploaded as supplementary information.
