# Supplementary material for: Knowledge of HbA1c and LDL‐C treatment goals, subjective level of disease‐related information and information needs in patients with atherosclerotic cardiovascular disease
Source: Clin Cardiol. 2022 Nov 30;46(2):223–31. doi: 10.1002/clc.23948 (PMC9933116; doi:10.1002/clc.23948)
Supplement: Supplementary file 1 — Supplementary information. [file CLC-46-223-s001.pdf]

SUPPLEMENTARY

Supplementary Table 1 –Symmetry tables

**a) HbA1c * LDL-C treatment goal attainment** **				
		**Attainment of LDL-C goal**		**Total**
		No	Yes	
**Attainment of HbA1c goal**	No	57 (28.1%)	23 (11.3%)	80 (39.4%)
	Yes	65 (32.0%)	58 (28.6%)	123 (60.6%)
**Total**		122 (60.1%)	81 (39.9%)	203
**b) HbA1c * LDL-C treatment goal knowledge** **				
		**Knowledge of LDL-C goal**		**Total**
		No	Yes	
**Knowledge of HbA1c goal**	No	98 (46.7%)	1 (0.4%)	99 (47.1%)
	Yes	107 (51.0%)	4 (1.9%)	111 (52.9%)
**Total**		205 (97.6%)	5 (2.4%)	210 (100%)
**c) HbA1c * LDL-C serum level knowledge** **				
		**Knowledge of LDL-C serum level**		**Total**
		No	Yes	
**Knowledge of HbA1c serum level**	No	61 (29.1%)	7 (3.3%)	68 (32.4%)
	Yes	112 (53.3%)	30 (14.3%)	142 (67.6%)
**Total**		173 (82.4%)	37 (17.6%)	210 (100%)

Supplementary Table 1: Tables (n (% of total)) of symmetry analysis using McNemar's test: a) attainment of glycated hemoglobin A1c (HbA1c) treatment goal vs. attainment of low-density lipoprotein cholesterol (LDL-C) treatment goal, n=203; b) knowledge of HbA1c vs. LDL-C treatment

Supplementary Table 2 – Associated factors of knowledge of HbA1c and LDL-C treatment goals

Knowledge of HbA1c treatment goal[a]			
**Variable**	**Odds ratio**	**95% confidence interval**	**p-value**
Age (years)	0.99	0.96-1.02	0.55
Sex (male/female)	1.35	0.70-2.60	0.37
Highest level of education (no->lower secondary->higher secondary-> university degree)	1.32	1.01-1.72	**0.04**
Patient participation preference (passive-> collaborative->active role)	1.20	0.83-1.73	0.33
Summed subjective level of patient knowledge: Topics of DM (1-24 points)	1.15	1.07-1.24	**<0.01**
**Knowledge of LDL-C treatment goal[b]**			
**Variable**	**Odds ratio**	**95% confidence interval**	**p-value**
Age (years)	1.08	0.94-1.24	0.28
Sex (male/female)	0.82	0.74-9.03	0.87
Highest level of education (no->lower secondary->higher secondary-> university degree)	2.32	1.07-5.03	**0.03**
Patient participation preference (passive-> collaborative->active role)	0.31	0.05-1.80	0.19
Summed subjective level of patient knowledge: Topics of ASCVD (1-24 points)	1.14	0.89-1.47	0.31

Supplementary Table 2: Associated factors of knowledge glycated hemoglobin A1c (HbA1c) and low-density lipoprotein cholesterol (LDL-C) treatment goals by multivariate logistic regression; n=210, no missings; DM=diabetes mellitus; ASCVD=atherosclerotic cardiovascular disease; [a]Hosmer-Lemeshow for goodness of fit of the model $X^2=13.45$, $df=8$, p=0.09; [b]Hosmer-Lemeshow for goodness of fit of the model $X^2=2.96$, $df=8$, p=0.93.

Supplementary Figure 1 – HbA1c and LDL-C serum levels

a) HbA1c levels

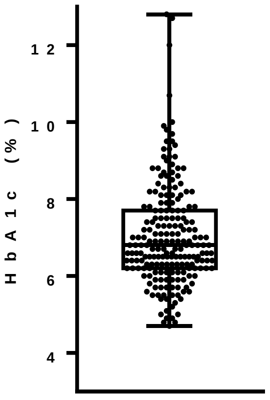

b) LDL-C levels

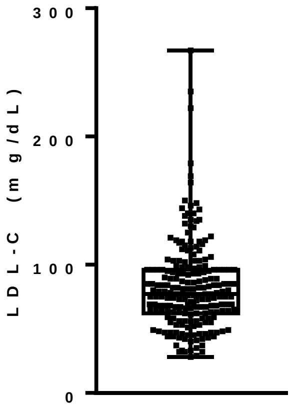

Supplementary Figure 1: Blood serum levels of (a) HbA1c and (b) LDL-C in all patients (n=203) with valid measurement of both parameters (scatterplots).

Supplementary Figure 2 – Correlations of summed subjective level of information and serum levels of HbA1c and LDL-C

a)

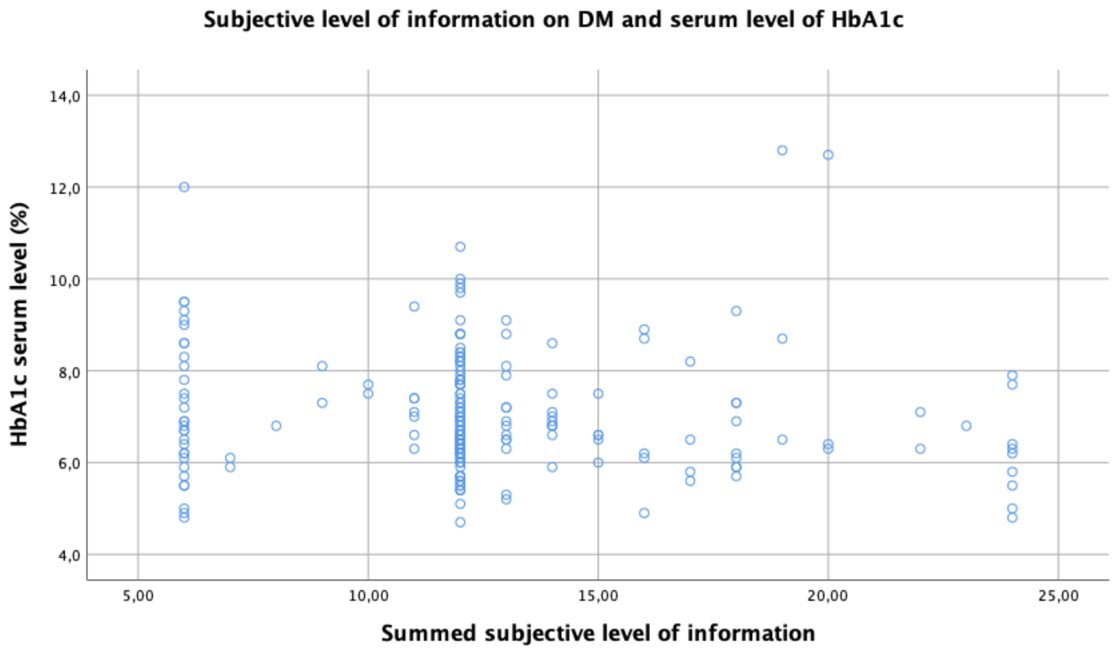

b)

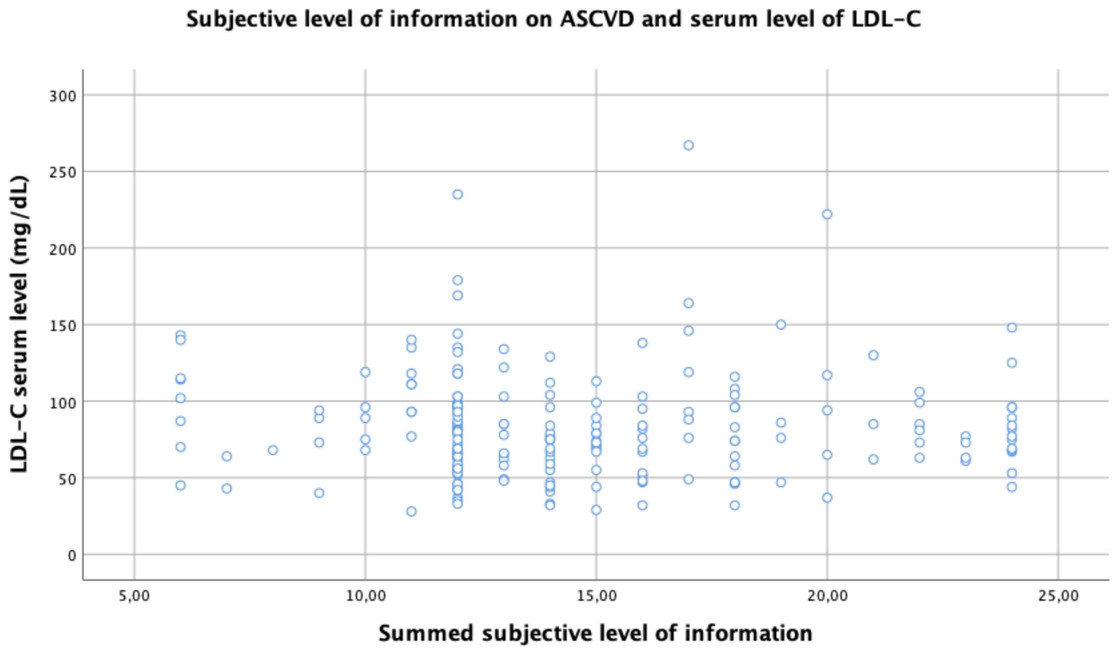

Supplementary Figure 2: (a) Correlation of summed subjective level of disease-related information on topics of diabetes mellitus (DM) with serum level of glycated hemoglobin A1c (HbA1c): Spearman's $\rho=0.11$, $p=0.14$; (b) correlation of summed subjective level of disease-related information on topics of atherosclerotic cardiovascular disease (ASCVD) with serum level of low-density lipoprotein cholesterol

(LDL-C): Spearman's $\rho=0.05$, $p=0.47$; low values indicating high levels of summed subjective level of information; $n=203$.

QUESTIONNAIRE

1. Which **role** would you like to obtain **in treatment** of your disease. Please select one of the following.

- ◻ I prefer to make the final decision about what treatment I will receive.
- ◻ I prefer to make the final selection of my treatment after seriously considering my doctor's opinion.
- ◻ I prefer that my doctor and I share responsibility for deciding which treatment is best for me.
- ◻ I prefer that my doctor makes the final decision about which treatment will be used, but seriously considers my opinion.
- ◻ I prefer to leave all decisions regarding my treatment to my doctor.

2. How well **informed** are you on the following **topics of your cardiovascular disease**?

					**Would you currently like more information on the topic?**	
**Causes of disease**						
	◻ very well	◻ well	◻ not well	◻ not informed at all	◻ yes	◻ no
**Course of disease**						
	◻ very well	◻ well	◻ not well	◻ not informed at all	◻ yes	◻ no
**Long-term complications**						
	◻ very well	◻ well	◻ not well	◻ not informed at all	◻ yes	◻ no
**Treatment and therapy**						
	◻ very well	◻ well	◻ not well	◻ not informed at all	◻ yes	◻ no
**Lifestyle adjustment, health promotion, and prevention**						
	◻ very well	◻ well	◻ not well	◻ not informed at all	◻ yes	◻ no
**Support, helplines, and information sources**						
	◻ very well	◻ well	◻ not well	◻ not informed at all	◻ yes	◻ no

3. How was your blood **LDL-cholesterol** (also known as **‚bad cholesterol'** or **LDL**) when **last measured**?

Please state a **value:**______________ OR **I don't know**: ◻

4. Which value should your **LDL-Cholesterol** ideally **not exceed**?

Please state a **value:**______________ OR **I don't know**: ◻

5. How well **informed** are you on the following **topics of your diabetes mellitus**?

					**Would you currently like more information on the topic?**	
**Causes of disease**						
	◻ very well	◻ well	◻ not well	◻ not informed at all	◻ yes	◻ no
**Course of disease**						
	◻ very well	◻ well	◻ not well	◻ not informed at all	◻ yes	◻ no
**Long-term complications**						
	◻ very well	◻ well	◻ not well	◻ not informed at all	◻ yes	◻ no
**Treatment and therapy**						
	◻ very well	◻ well	◻ not well	◻ not informed at all	◻ yes	◻ no
**Lifestyle adjustment, health promotion, and prevention**						
	◻ very well	◻ well	◻ not well	◻ not informed at all	◻ yes	◻ no
**Support, helplines, and information sources**						
	◻ very well	◻ well	◻ not well	◻ not informed at all	◻ yes	◻ no

6. How was your **HbA1c** in % (also known as **long-term blood glucose value**) when **last measured**?

Please state a **value:**______________ OR **I don't know**: ◻

7. Which value should your **HbA1c** in % ideally **not exceed**?

Please state a **value:**______________ OR **I don't know**: ◻

8. What is your **highest level of education**?

◻ no degree
◻ main/public school
◻ middle school
◻ high school diploma
◻ university
